# A Small Molecule Inhibitor of ETV1, YK-4-279, Prevents Prostate Cancer Growth and Metastasis in a Mouse Xenograft Model

**DOI:** 10.1371/journal.pone.0114260

**Published:** 2014-12-05

**Authors:** Said Rahim, Tsion Minas, Sung-Hyeok Hong, Sarah Justvig, Haydar Çelik, Yasemin Saygideger Kont, Jenny Han, Abraham T. Kallarakal, Yali Kong, Michelle A. Rudek, Milton L. Brown, Bhaskar Kallakury, Jeffrey A. Toretsky, Aykut Üren

**Affiliations:** 1 Lombardi Comprehensive Cancer Center, Georgetown University Medical Center, Washington, DC, United States of America; 2 The Sidney Kimmel Comprehensive Cancer Center, Johns Hopkins University, Baltimore, MD, United States of America; Florida International University, United States of America

## Abstract

**Background:**

The erythroblastosis virus E26 transforming sequences (*ETS*) family of transcription factors consists of a highly conserved group of genes that play important roles in cellular proliferation, differentiation, migration and invasion. Chromosomal translocations fusing ETS factors to promoters of androgen responsive genes have been found in prostate cancers, including the most clinically aggressive forms. ERG and ETV1 are the most commonly translocated ETS proteins. Over-expression of these proteins in prostate cancer cells results in a more invasive phenotype. Inhibition of ETS activity by small molecule inhibitors may provide a novel method for the treatment of prostate cancer.

**Methods and Findings:**

We recently demonstrated that the small molecule YK-4-279 inhibits biological activity of ETV1 in fusion-positive prostate cancer cells leading to decreased motility and invasion *in-vitro*. Here, we present data from an *in-vivo* mouse xenograft model. SCID-beige mice were subcutaneously implanted with fusion-positive LNCaP-luc-M6 and fusion-negative PC-3M-luc-C6 tumors. Animals were treated with YK-4-279, and its effects on primary tumor growth and lung metastasis were evaluated. YK-4-279 treatment resulted in decreased growth of the primary tumor only in LNCaP-luc-M6 cohort. When primary tumors were grown to comparable sizes, YK-4-279 inhibited tumor metastasis to the lungs. Expression of ETV1 target genes MMP7, FKBP10 and GLYATL2 were reduced in YK-4-279 treated animals. ETS fusion-negative PC-3M-luc-C6 xenografts were unresponsive to the compound. Furthermore, YK-4-279 is a chiral molecule that exists as a racemic mixture of R and S enantiomers. We established that (S)-YK-4-279 is the active enantiomer in prostate cancer cells.

**Conclusion:**

Our results demonstrate that YK-4-279 is a potent inhibitor of ETV1 and inhibits both the primary tumor growth and metastasis of fusion positive prostate cancer xenografts. Therefore, YK-4-279 or similar compounds may be evaluated as a potential therapeutic tool for treatment of human prostate cancer at different stages.

## Introduction

Chromosomal rearrangement is a common mechanism driving oncogenesis in sarcomas and hematologic malignancies [1]. Recently, fusions involving the erythroblastosis virus E26 transforming sequences (*ETS*) family of transcription factors have been discovered in prostate cancer tumors [2]. The *ETS* family of transcription factors is a highly conserved group of genes consisting of 27 members, many of which have been shown to play important roles in disease initiation, progression, differentiation, migration, invasion and angiogenesis [3], [4]. ETS proteins share significant homology with each other and contain a C-terminal *ETS* domain that is involved in DNA-binding and a N-terminal PNT domain involved in protein interactions [5]. Chromosomal rearrangements involving ETS factors in prostate cancer cells place them under direct regulation of androgen responsive gene promoters, thereby activating their expression in response to androgens. Unlike the protein products of chromosomal translocations in leukemias and sarcomas, gene rearrangements in prostate cancer do not create chimeric fusion proteins. Instead, most chromosomal translocations and gene rearrangements involving ETS factors in prostate cancer result in expression of a full length or nearly full length ETS family proteins.

Translocations involving ERG and ETV1 constitute the majority of ETS rearrangements found in prostate cancer. Whereas ERG is predominantly fused to TMPRSS2 promoter, ETV1 can be rearranged with the 5′ region of several genes, such as TMPRSS2, SLC45A3 and HNRPA2B1 [2], [6]. *ETV1* translocation results in the expression of full-length or N-terminal truncated ETV1 [7]. Over-expression of ETV1 in benign prostatic epithelial cell-lines results in the induction of a subset of genes involved in migration and invasion [6]. ETV1 also increases expression of AR target genes, as well as genes involved in steroid biosynthesis and metabolism. Co-operation with other oncogenic events, such as PTEN loss, predisposes ETV1 expressing prostate cells to evolve into a more aggressive disease phenotype [8], [9]. Studies in murine models suggest that ETV1 expression is an underlying cause of prostate cancer initiation. ETV1 transgenic mice develop prostatic intraepithelial neoplasia. In addition, combining ETV1 expression with pre-existing genomic lesions, such as PTEN loss, results in development of invasive adenocarcinoma [10], [11].

We recently reported that YK-4-279, an inhibitor of EWS-FLI1 oncoprotein in Ewings sarcoma, also inhibits ERG and ETV1 activity in prostate cancer cells *in-vitro*, resulting in reduced migratory and invasive phenotypes [12], [13]. Based upon our prior *in-vitro* investigations, we tested the anti-metastatic ability of YK-4-279 in a mouse xenograft model. Animals treated with YK-4-279 had reduced tumor growth and reduced metastasis of the tumor from primary site to lungs. We also demonstrate that the effects of YK-4-279 on ETV1 and prostate cancer cell lines are enantiospecific and (S)-YK-4-279 enantiomer is the active component confirming similar findings in other tumor models [14].

## Results and Discussion

### YK-4-279 is a small molecule antagonist of ETV1

We initially focused on evaluating the effects of YK-4-279 on tumor metastasis *in-vivo*, since our *in-vitro* experiments with prostate cancer cell lines suggested that it primarily inhibits motility and invasion [13]. To test the efficacy of YK-4-279 *in-vivo*, we utilized a mouse xenograft model [15], [16]. LNCaP-luc-M6 and PC-3M-luc-C6 prostate cancer cell lines are generated by stable transfection of parental LNCaP and PC-3 cells with a vector expressing luciferase gene. The cells are subcutaneously injected below the dorsal flank in 8-10 weeks old SCID/beige male mice. Lung metastasis can be seen as early as 6-7 weeks following tumor implantation in these animals [15], [16].

**Figure 1:**
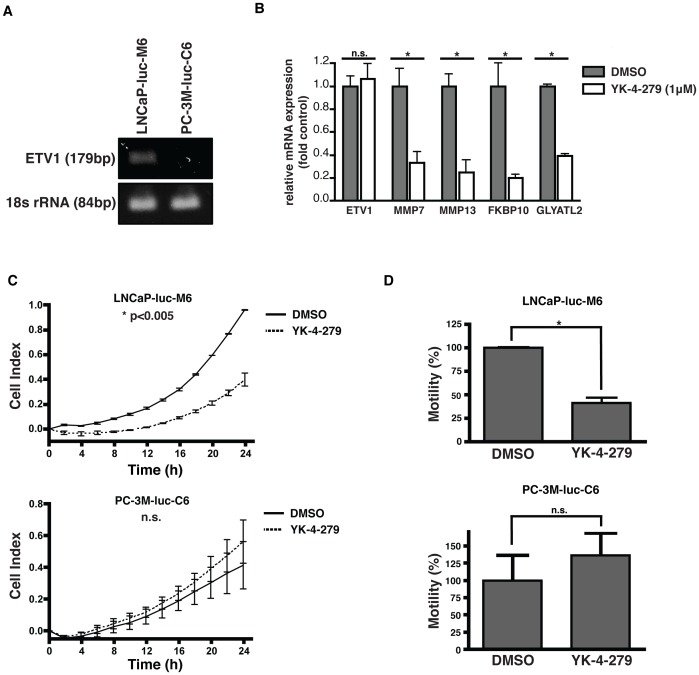
YK-4-279 is a small molecule inhibitor of ETV1. a) Genomic DNA from prostate cells was analyzed for ETS rearrangement status by performing PCR using rearrangement specific primers. LNCaP-luc-M6 cells harbored ETV1 rearrangement whereas PC-3M-luc-C6 cells were fusion-negative. b) LNCaP-luc-M6 cells were treated with 1 µM YK-4-279 for 48 hours and ETV1 target gene levels were evaluated by real-time quantitative PCR. YK-4-279 treatment resulted in decreased gene expression of MMP7, MMP13, GLYATL2 and FKBP10 without significant reduction in ETV1 levels. *; p<0.01, n.s.; not-significant, unpaired student's t-test. c) LNCaP-luc-M6 and PC-3M-luc-C6 were pre-treated with 1 µM YK-4-279 for 48 hours. An electrical impedance based chemotaxis assay was used to monitor cell migration in the presence of YK-4-279 towards the lower chamber with 10% FBS gradient. YK-4-279 inhibited the migration of LNCaP-luc-M6 but not PC-3M-luc-C6 cells. *; p<0.005, n.s.; not-significant, unpaired student's t-test. d) Motilities of cells at the end of 24 hr period were calculated based on their relative cell index values. *; p<0.01, n.s.; not-significant, unpaired student's t-test.

We previously demonstrated that inhibition of ETV1 biological activity in LNCaP cells results in decreased invasion and migration without affecting the growth in culture [13]. The cell lines used in current study were commercially acquired from a different source than our earlier work and underwent selective pressure to obtain stable luciferase expressing clones. We first validated the effect of YK-4-279 on these cells before proceeding to *in-vivo* models. LNCaP cells contain a genetic translocation where the entire ETV1 locus is inserted in the last intron of the prostate-specific MIPOL1 region on chromosome 14. We verified the presence of ETV1 translocation in LNCaP-luc-M6 cells by genomic DNA PCR using primers flanking the recombination site (Fig. 1a). ETV1 rearrangement was exclusive to LNCaP-luc-M6 cells and not present in the PC-3M-luc-C6 cells. Thus, the PC-3M-luc-C6 cell line was selected as a negative control for our studies.

We treated LNCaP-luc-M6 cells with a sub lethal dose (1 µM) of YK-4-279 for 48 hours and evaluated expression of endogenous ETV1 target genes by real time quantitative PCR. We focused on known ETV1 targets that are implicated in prostate pathogenesis [17]-[19]. Exposure of LNCaP-luc-M6 cells to 1 µM YK-4-279 resulted in significantly reduced mRNA levels of several ETV1 target genes, including MMP7, MMP13, FKBP10 and GLYATL2, without affecting the expression of ETV1 (Fig. 1b).

Next, we performed an electric impedance-based chemotaxis assay to determine the effects of YK-4-279 on motility of LNCaP-luc-M6 and PC-3M-luc-C6 cells. This technique involves the use of a Boyden Chamber-like setup with microelectronic sensors integrated under a microporous polyethylene terephthalate (PET) membrane. The sensors record electrical impedance as cells migrate from the upper chamber, through the membrane, and into the bottom chamber in response to a chemoattractant. This technique permits real-time monitoring of cell migration as increases in electrical impedance correlate with increasing number of migrated cells to the bottom chamber. YK-4-279 treatment of LNCaP-luc-M6 cells resulted in a significant decrease in cell migration, while no effect was observed on the motility of the negative control cell-line, PC-3M-luc-C6 (Fig. 1c and 1d). These findings confirmed that commercially available LNCaP-luc-M6 and PC-3M-luc-C6 cells had the same phenotypes and YK-4-279 response profiles as LNCaP and PC-3 cells that we used in our earlier studies.

### YK-4-279 inhibits tumor growth

*In-vivo* YK-4-279 treatment experiments were done in two different formats: 1) Early treatment experiments, where YK-4-279 administration was started the day after xenograft implantation. 2) Late treatment experiments, where YK-4-279 administration started only after the primary xenograft tumor reached to a palpable size (∼200 mm^3^). These two approaches allowed us to evaluate the effects of YK-4-279 on tumor up take, growth and lung metastasis both prior to formation of well-established tumors as well as after palpable tumor formation.

**Figure 2:**
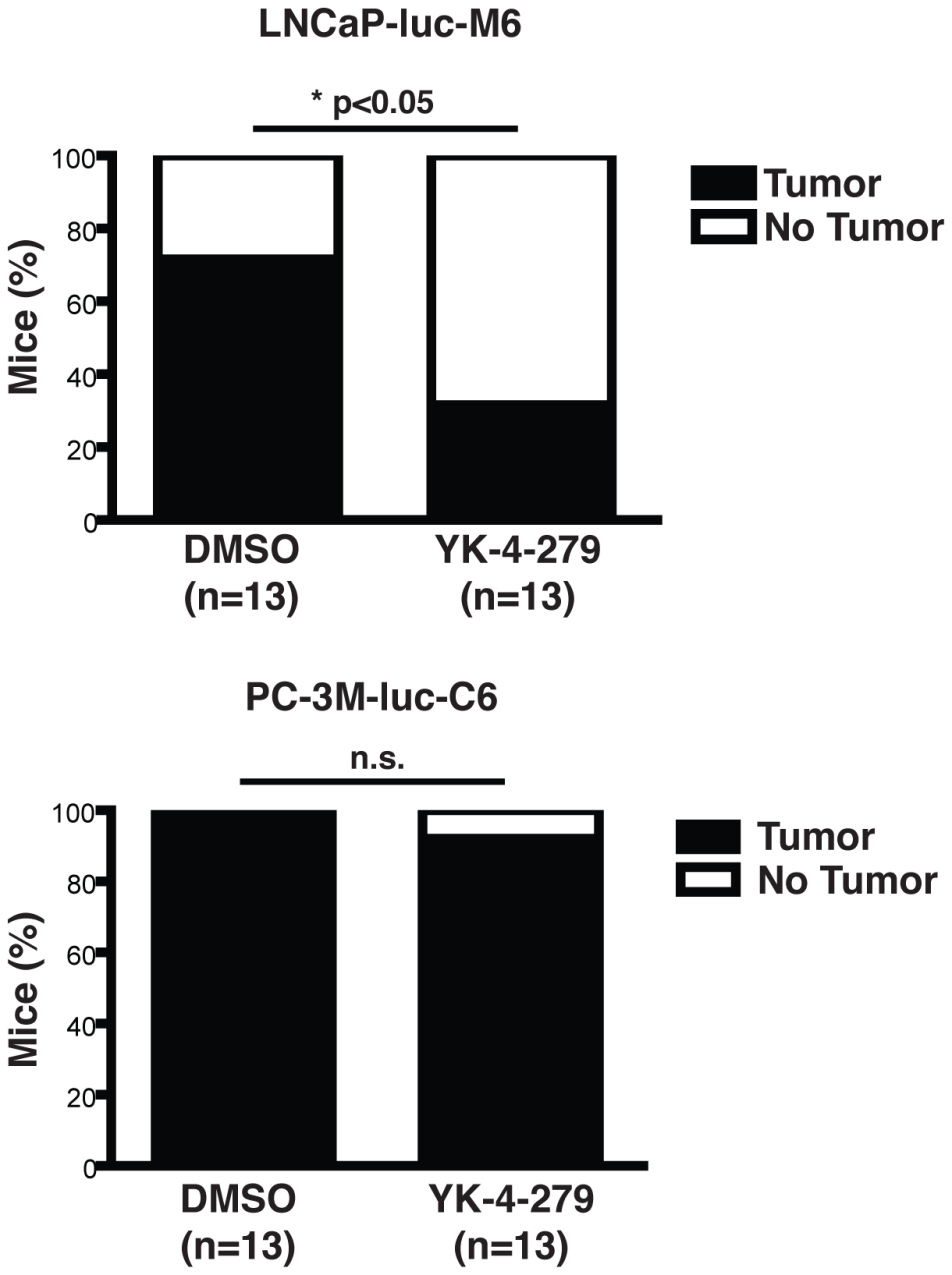
YK-4-279 reduced tumor uptake when administered prior to tumor formation. Prostate xenografts were established by subcutaneously injecting cells below the dorsal flank in 8-10 weeks old SCID/beige male mice. Animals were treated with 75 mg/kg body weight YK-4-279 thrice weekly, starting the day after xenograft injections. LNCaP-luc-M6 animals treated with compound displayed decreased tumor formation (4/13) compared to vehicle control (9/13). PC-3M-luc-C6 animals did not display significant difference in tumor formation between compound treated (12/13) and vehicle control (13/13) animals. *; p<0.05, n.s.; not-significant, unpaired student's t-test.

**Figure 3:**
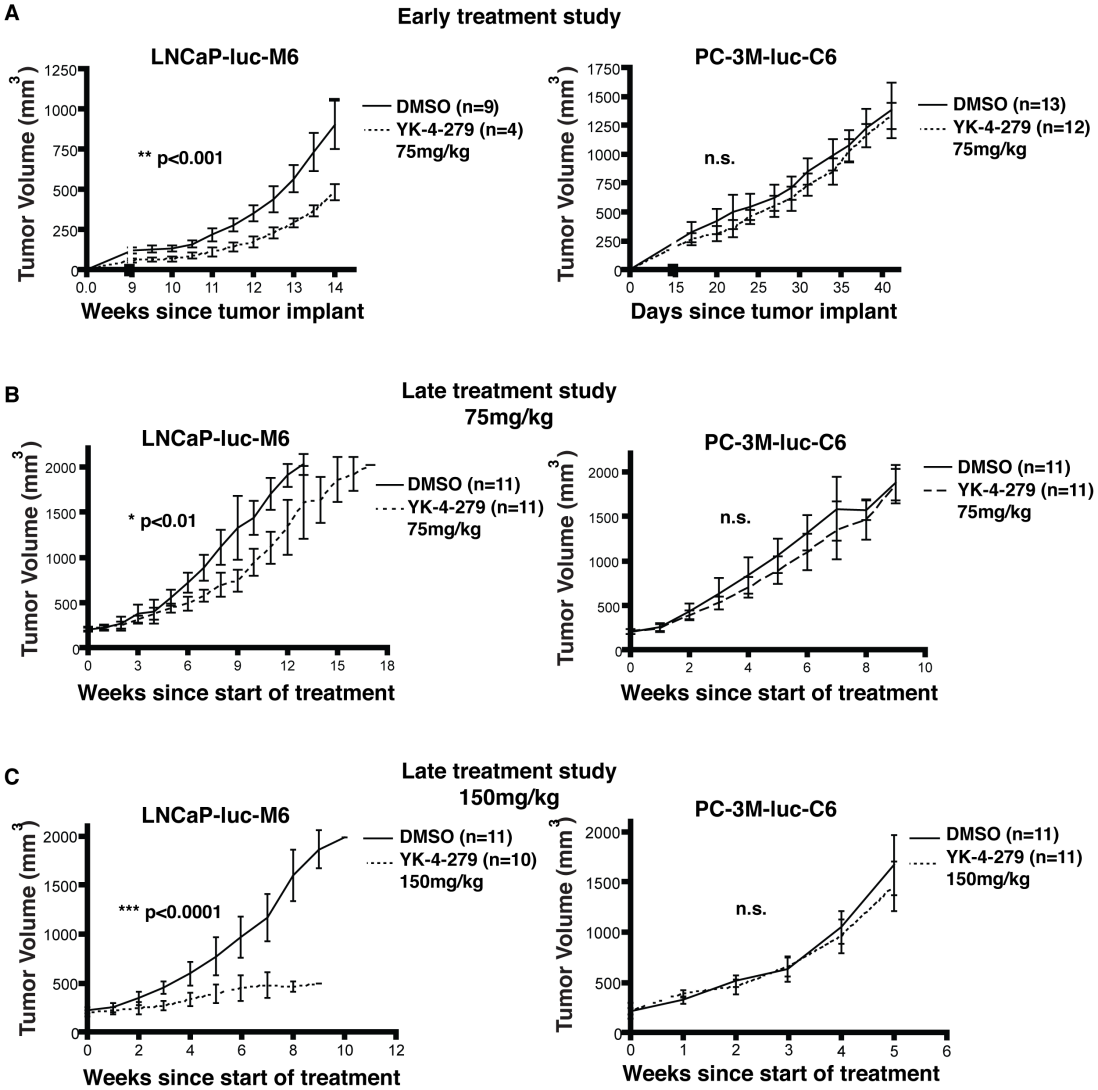
YK-4-279 reduces tumor growth in LNCaP-luc-M6 mice. a) SCID/beige mice were subcutaneously injected with LNCaP-luc-M6 or PC-3M-luc-C6 cells below the dorsal flank. In the early treatment study group, animals were injected with 75 mg/kg YK-4-279 starting the day after xenograft injection. b) Another set of animals started receiving YK-4-279 treatment once the tumors were palpable (∼200 mm^3^). These animals were further divided in to 2 separate cohorts: one group was treated three times a week with 75 mg/kg YK-4-279 (late treatment study low dose). c) Another group was treated 5 times a week with 150 mg/kg compound (late treatment study high dose). Tumor volumes were measured weekly. YK-4-279 reduced tumor growth in LNCaP-luc-M6 animals, but not in PC-3M-luc-C6 animals. *; p<0.01, **; p<0.001, ***; p<0.0001, n.s.; not-significant.

We established prostate xenografts by subcutaneously injecting LNCaP-luc-M6 or PC-3M-luc-C6 cells into the dorsal flank of SCID/beige mice. In the early treatment study, we started intraperitoneal drug treatment with 75 mg/kg YK-4-279 or vehicle control the day after tumor cell injection. Animals were treated 3 times per week and tumor volumes measured weekly. The study was terminated after 14 weeks for the LNCaP-luc-M6 group and 6 weeks for the PC-3M-luc-C6 group due to the relatively faster growth rate of PC-3M-luc-C6 cells. While only 4 of the 13 mice that were subcutaneously injected with LNCaP-luc-M6 cells and treated with YK-4-279 developed tumors, in stark contrast, 9 of the 13 animals in the vehicle control group developed tumors. No such difference was present in the fusion-negative PC-3M-luc-C6 cohort (Fig. 2). In animals that developed tumors, there was a significant reduction in tumor size in YK-4-279 treated group compared to DMSO control. Reduction in tumor size was only present in the LNCaP-luc-M6 group and not observed with PC-3M-luc-C6 xenografts (Fig. 3a). Our prior *in-vitro* investigations revealed that ETV1 inhibition by YK-4-279 leads to reduced motility and invasion without affecting cell growth and survival. Hence, we did not expect to see a difference in tumor uptake or primary tumor growth rate in these animals. The early treatment experiment was designed to measure lung metastasis, and all animals were euthanized at the predetermined endpoint (6 weeks for PC-3M-luc-C6 and 14 weeks for LNCaP-luc-M6) to harvest tissues for further analysis.

The late treatment studies started with xenograft implantation and close follow up. When the animals showed a palpable tumor (∼200 mm^3^), they were randomized to YK-4-279 and vehicle control (DMSO) groups. The end-point for the late treatment study was selected as the primary tumor size reaching 2 cm^3^ in all groups so that metastatic burden between groups could be evaluated with equal primary tumor size in all groups. Furthermore, the late treatment study was repeated twice with two different YK-4-279 doses; 75 mg/kg YK-4-279 three times a week and 150 mg/kg YK-4-279 five times a week.

Primary tumor growth was significantly reduced in late treatment study as well (Fig. 3b). This difference was enhanced when the drug dose and frequency were increased (Fig. 3c). In the high dose group, however, animals began showing signs of hyperventilation and lethargy, prompting a dose reduction to 150 mg/kg given 4 days a week after week 4 and 3 days a week after week 8. Drug treatment did not affect the growth rate of fusion-negative PC-3M-luc-C6 xenografts at either dose.

A group of primary tumor samples were also evaluated for histopathological parameters (Fig. S1). Areas of tumor necrosis were identified in H&E stained slides. Amount of cell proliferation was determined by Ki67 immunohistochemistry and amount of apoptosis was determined by TUNNEL staining. LNCaP tumors in general showed an increase in necrosis in response to YK-4-279 treatment in high dose group (150 mg/kg). Similarly we observed a reduction in Ki67 staining in LNCaP tumors in the treatment group. However, these differences in LNCaP tumors were not statistically significant (Fig S2). There was no appreciable difference in PC3 tumors for either assay.

### YK-4-279 inhibits lung metastasis in LNCaP-luc-M6 xenograft animals

**Figure 4:**
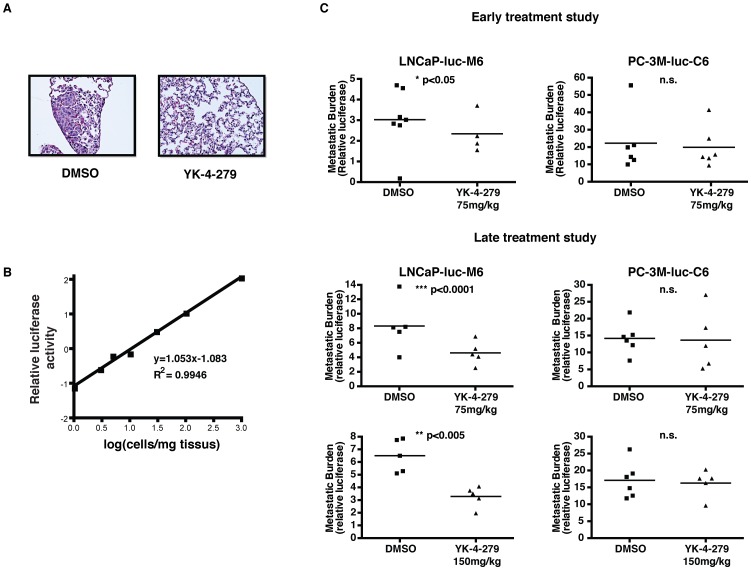
YK-4-279 inhibits lung metastasis in LNCaP-luc-M6 xenograft animals. a) H&E stained lung sections showing a micro-metastatic lesion in DMSO treated LNCaP-luc-M6 animals. b) A standard curve was constructed to measure the detection limit of the luciferase assay. The assay is extremely sensitive, allowing the detection of a single prostate cancer cell per milligram lung tissue. c) Lungs were harvested from xenograft animals 15 minutes after the last compound or vehicle treatment. Protein lysates were obtained from the tissues and used to perform a luciferase assay. Results were normalized to tissue weight. Compound treated LNCaP-luc-M6 xenograft animals displayed significantly reduced lung metastasis compared to vehicle controls. PC-3M-luc-C6 lung metastasis was unaffected by compound treatment. *; p<0.05, **; p<0.005, ***; p<0.0001, n.s.; not-significant, unpaired student's t-test.

We developed a total cell-lysate based assay that took advantage of the luciferase protein expression to accurately quantify the metastasis of prostate cancer cells to the lungs. The assay involves extraction of protein lysates from lungs, followed by luciferase measurements. Metastasis of LNCaP-luc-M6 and PC-3M-luc-C6 cells to the lungs results can be demonstrated in H&E stained lung sections when they are big enough (Fig. 4a). However, this approach is not quantitative and may miss micro metastases especially in unsectioned portions of the organ left in the paraffin block. It is crucial to have an assay sensitive enough to detect the presence of even the small number of prostate tumor cells in the lungs. We constructed a standard curve by combining serial dilutions of luciferase expressing prostate cancer cells grown in culture with lung tissues from healthy mice (Fig. 4b). Using this assay, we were able to detect as little as 1 cell per milligram lung tissue. Considering an average lung mass of 140 mg, this assay has a lower detection limit of 140 prostate cancer cells per lung [20]. Since luciferase expression is the primary method used to quantify tumor metastasis, we performed an *in-vitro* luciferase assay, using doses of YK-4-279 that suppress invasion, to demonstrate that compound treatment dose not affect expression of luciferase in LNCaP-luc-C6 or PC-3M-luc-C6 cells. We also measured luciferase expression in primary tumors extracted from animals treated with the highest dose of YK-4-279 or vehicle control. Compound treatment did not affect luciferase expression either *in-vitro* or *in-vivo* (Fig. S3).

We then performed the same luciferase assay on the lungs of xenograft carrying animals treated with YK-4-279 or vehicle control. In all 3 experiments (early treatment study, late treatment study low dose, late treatment study high dose), compound treatment resulted in a significant reduction in lung metastasis in LNCaP-luc-M6 xenograft animals, but not in PC-3M-luc-C6 animals (Fig. 4c). We also used a PCR based assay to quantify lung metastasis in the late treatment study high dose cohort using human specific primers for the Ribonuclease P RNA component H1 (RPPH1) and mouse specific primers that detect the transferrin receptor gene (Tfrc). This assay confirmed our earlier observations revealing significantly reduced lung metastasis in the YK-4-279 treated LNCaP-luc-M6 group (Fig. S4) and validated that luciferase measurement in lung tissue was a reliable method. In two experiments (early treatment study and late treatment study high dose), lungs were harvested from LNCaP-luc-M6 xenograft animals that displayed reduced primary tumor sizes in the treatment group at the time of tissue acquisition (Fig. 3a and Fig. 3c). Hence, it is possible that the differences in lung metastasis may be a direct result of smaller tumor volumes in treatment groups. However, the late treatment study low dose group had similar primary tumor volumes (2 cm^3^) when tissues were harvested from the animals (Fig. 3b). YK-4-279 reduced lung metastasis in these animals as well, suggesting that drug treatment affects tumor metastasis by inhibiting ETV1 activity, independent of the primary tumor size.

**Figure 5:**
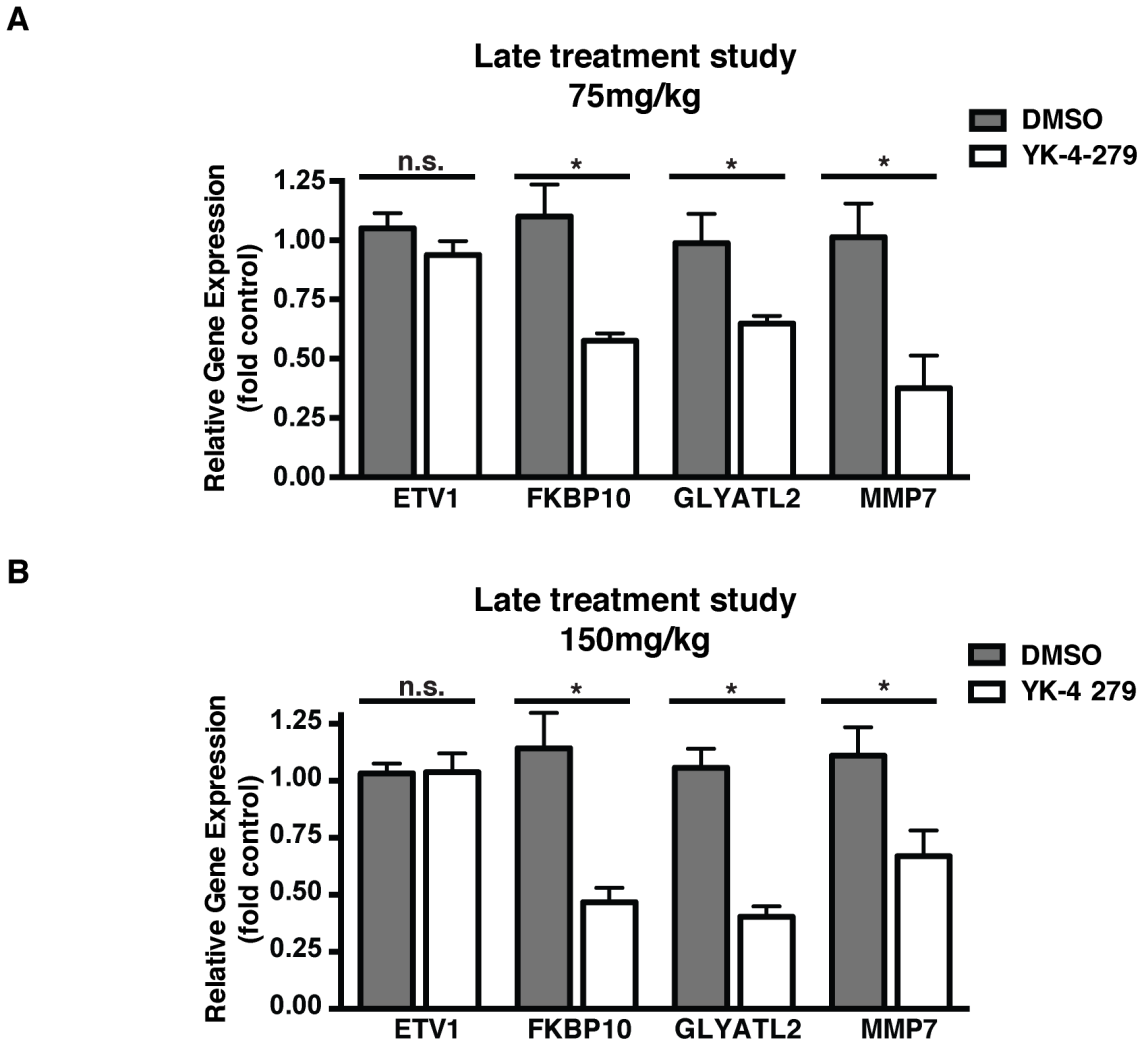
YK-4-279 inhibits ETV1 target gene expression in-vivo. a) RNA was extracted from tumors of compound and vehicle treated LNCaP-luc-M6 animals (late treatment study low dose) 15 minutes after the last injection. Gene expression levels were determined by quantitative real-time PCR. Results were normalized to 18s rRNA expression. Experiments were performed in triplicates with 5 mice analyzed per group. YK-4-279 treatment resulted in decreased gene expression of MMP7, GLYATL2 and FKBP10 without significant reduction in ETV1 levels. *; p<0.05, n.s.; not-significant, unpaired student's t-test. b) ETV1 target gene expression levels in late treatment study high dose group. *; p<0.05, n.s.; not-significant, unpaired student's t-test.

We also evaluated ETV1 target gene expression in primary tumors upon YK-4-279 treatment. ETV1 inhibition by YK-4-279 resulted in reduced MMP-7, FKBP10 and GLYATL2 expression, without affecting ETV1 expression levels (Fig. 5a and Fig. 5b). To determine whether differences in drug response between LNCaP-luc-M6 and PC-3M-luc-C6 animals is a factor of differential tumor penetration of the compound between the two cohorts, we measured the concentration of YK-4-279 in the plasma and tumors of animals following the last dose of YK-4-279. LNCaP-luc-M6 mice displayed an average concentration of 106.9±64.1 µg/mL YK-4-279 in the plasma and 27.8±14.5 µg/g in the tumor for a tumor:plasma ratio of 0.30±0.20. PC-3M-luc-C6 animals demonstrated 174.8±53.5 µg/mL YK-4-279 in the plasma and 23.3±12.3 µg/g in the tumor for a tumor:plasma ratio of 0.15±0.10. There was no statistically significant difference in the penetration of YK-4-279 between the two groups when compared by a chi-square test.

### Enantiospecific effects of YK-4-279

**Figure 6:**
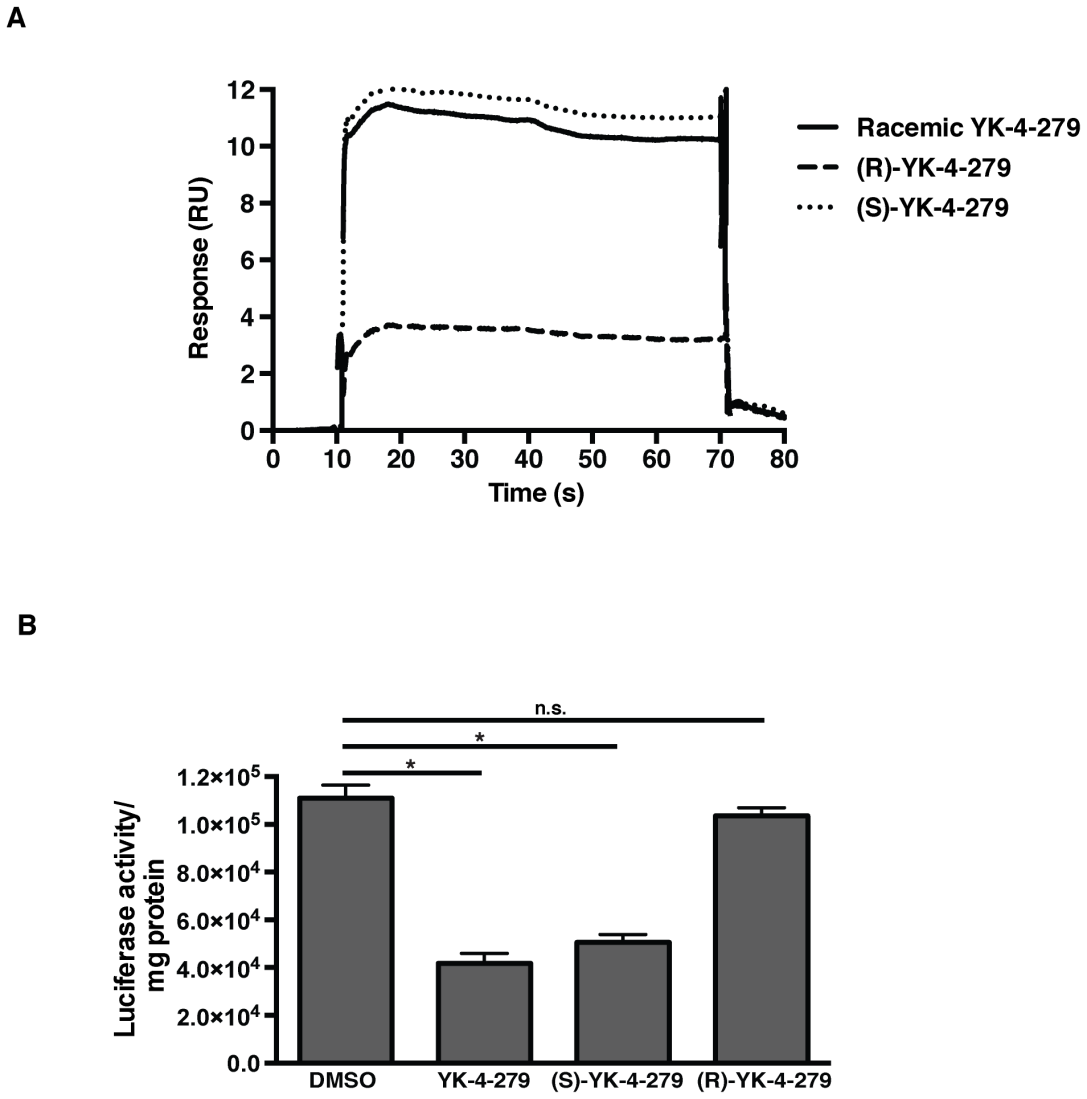
(S)-YK-4-279 is the active enantiomer of YK-4-279. a) Racemic YK-4-279, (R)-YK-4-279 and (S)-YK-4-279 were injected over a Biacore chip surface containing recombinant ETV1. Racemic YK-4-279 and the S-enantiomer bound to ETV1 whereas the R-enantiomer had a lower binding affinity to ETV1. b) A luciferase assay was performed in Cos-7 cells co-transfected with ETV1 and an Id-2 reporter luciferase construct. Id-2 promoter activity was decreased upon treatment with racemic YK-4-279 and (S)-YK-4-279. *; p<0.0005, n.s.; not-significant, unpaired student's t-test.

YK-4-279 has a chiral center and the racemic compound can be separated into its constituent R and S enantiomers by high-pressure liquid chromatography (HPLC), or each enantiomer can be synthesized individually. In Ewings sarcoma models, the S-enantiomer has been established as the active component that inhibits EWS-FLI1, whereas the R-enantiomer has virtually no specific activity [14], [21]. We tested whether the same phenomenon is true for inhibition of ETV1 in prostate cancer cells. Surface plasmon resonance (SPR) experiments were performed to determine the binding of racemic YK-4-279 and each individual enantiomer to ETV1. Compounds were injected over a Biacore chip surface containing recombinant ETV1. Racemic YK-4-279 and the S-enantiomer bound to ETV1 whereas the R-enantiomer showed a weaker binding to ETV1 (Fig. 6a). We then evaluated YK-4-279 for its effect on ETV1 transcriptional activity using a transiently transfected luciferase reporter construct, which contains a minimal Id2 promoter region with two binding sites for ETV1. Co-transfection of ETV1 and Id2 reporter in COS-7 cells resulted in an increase in luciferase activity. Promoter activity was reduced by treatment of the cells with racemic YK-4-279 and (S)-YK-4-279. However, (R)-YK-4-279 did not inhibit ETV1 transcriptional activity (Fig. 6b).

Chiral discrimination between enantiomers is an important feature of many drug like molecules, as single active enantiomers can provide greater selectivity for their biological targets, improve therapeutic index, and display better pharmacokinetics than the racemic mixture [22]. It can also reduce the total drug dose and decrease drug interactions and toxic side effects. When submitting a new drug for approval, the U.S. Food and Drug Administration (FDA) requires developers to justify the choice of using a racemic mixture over single-enantiomer formulations.

In our *in-vivo* experiments, LNCaP-luc-M6 tumor growth inhibition was greater at 150 mg/kg YK-4-279 compared to 75 mg/kg. However, animals receiving a higher drug dose could not be treated for longer than 10-12 weeks due to the manifestation of necrotic tumors, which required the animals to be euthanized. Thus, in addition to metastasis inhibition, YK-4-279 may have a direct effect on proliferation in ETS-positive prostate cancer cells. The only drawback of treating animals with higher doses of YK-4-279 was the appearance of drug toxicity symptoms that started at 4 weeks. In this study, we managed the symptoms by decreasing drug treatment frequency, thus giving the animals more recovery time between injections. However, in order to move this compound into the clinic, further investigation is required to improve the *in-vivo* efficacy and address the symptoms that arise at higher doses. We are currently exploring various dosing regimens and formulations to find the ideal treatment scenario that maximizes on-target effects while minimizing off-target drug toxicities. Due to its hydrophobic structure, the bioavailability of YK-4-279 is only 2%-15% when it is administered to mice by oral gavage [14]. In addition, recent pharmacokinetic experiments in our laboratory have shown that intra-peritoneal administrations of 75 mg/kg YK-4-279 initially leads to a steep rise in plasma concentrations, but is substantially cleared leading to ∼1 µM levels by 2 hours [14]. The pharmacokinetic properties of YK-4-279, along with the inability to deliver a sustainable high dose over time through bolus injections suggests the need to develop a continuous infusion model to ensure adequate drug delivery. We have tested this paradigm in an Ewing's sarcoma xenograft model in nude rats. These animals receive continuous drug infusion via a central venous catheter and show better response to YK-4-279 treatment than daily intra-peritoneal or intra-venous injections [21]. Further combining pharmacokinetic measurements, *in-vivo* modeling and laboratory studies will allow us to create an optimal drug delivery formulation that is suitable for clinical use. In addition, successful validation of (S)-YK-4-279 as the active component of YK-4-279 may allow drastically reduced treatment dose *in-vivo.*

A growing body of evidence suggests that ETS fusions function concomitantly with other genomic alterations in the initiation and maintenance of prostate malignancies. Androgen-inducible prostate-specific overexpression of ETV1 in transgenic mice induces prostatic intraepithelial neoplasia (PIN) but does not lead to carcinoma formation. Crossing these transgenic mice into a PTEN^+/-^ background or constitutively active prostate-specific PI3K/Akt pathway induces invasive carcinoma within 6 months, suggesting that ETS translocations co-operate with other genetic lesions to induce prostate cancer in humans [10], [11]. Recent findings have also identified Poly(ADP-Ribose) Polymerase (PARP), a key DNA repair protein, to interact with ETS factors in a DNA independent manner [23]. PARP1 is shown to be important for ETS protein function, and inhibition of PARP1 impairs ETS mediated tumorigenesis and cell invasion. PARP and PI3K/Akt pathway inhibitors such as olaparib, rucaparib, perifosine, and miltefosine are at an advanced state of clinical testing [24]–[26]. An important clinical advantage of these findings is the potential to combine YK-4-279 with other drugs to achieve a more robust and synergistic response, opening a wide spectrum of new strategies to target ETS factors.

Transcription factors have been historically considered difficult targets due to the complex regulation of their target genes, their lack of enzymatic activity and the widespread network of protein binding partners required for their function. However, the successful modulation of transcription factor function in several cancers has now revealed that this large and important class of proteins is indeed “druggable” [27]–[30]. Our data establishes YK-4-279 as a specific inhibitor of ETV1 transcriptional activity in fusion-positive prostate cancer cells, leading to decreased growth and metastatic dissemination of cells. Successful clinical application of this compound will be a useful therapeutic tool for the treatment of prostate cancer and inhibition of prostate cancer metastasis.

## Materials and Methods

### Cell Culture

LNCaP-luc-M6 and PC-3M-luc-C6 were purchased from PerkinElmer (Waltham, MA). Cells were maintained in RPMI media supplemented with 10% heat-inactivated FBS.

### mRNA isolation and qPCR

mRNA from cells growing in culture was isolated using TRIzol (Invitrogen). mRNA from animal tissues was extracted using RNAeasy mini kit (Qiagen, Venlo, Netherlands). cDNA was prepared using transcriptor first-strand cDNA synthesis kit (Roche, San Francisco, CA) according to manufacturer's protocol. qRT-PCR was carried out using SYBR green (Roche) on a Mastercycler realplex^4^ instrument (Eppendorf, New York, NY). Gene expression was normalized to 18s rRNA. Differences in gene expression were calculated using ΔΔCt method. A PCR profile of 90°C- 10 min: 1 cycle, 90°C- 30 sec, 55°C (varies)- 30 sec, 72°C- 1 min: 40 cycles, 72°C- 5 min: 1 cycle was used.

### Rearrangement status

Genomic DNA was isolated from PC-3M-luc-C6 and LNCaP-luc-M6 cells using Wizard genomic DNA extraction kit (Promega, Madison, WI) according to manufacturer's protocols. PCR was carried out using primers flanking rearrangement sites. Primer sequences are provided in Table S1.

### Chemotaxis

Chemotaxis was performed using CIM-16 plates in a RTCA DP instrument (ACEA Biosciences, San Diego, CA). CIM-16 plates consist of an upper and a lower chamber separated by a microporous polyethylene terephthalate (PET) membrane with microelectronic sensors integrated under the membrane. The sensors measure variations in electrical impedance to quantify cells migrating through the membrane and towards the chemoattractant. The bottom chamber of the CIM-16 plate was filled with 160 µL RPMI media containing 10% FBS as the chemoattractant. The upper chamber was assembled on top of the bottom chamber and the plate was allowed to incubate at 37°C for 1 hour. LNCaP-luc-M6 or PC-3M-luc-C6 cells were harvested and added to the upper chamber in 100 µL serum-free media. Impedance measurements were recorded at 15 minute intervals for 24 hours.

### Animal Studies

8-10 weeks old male SCID/beige mice were subcutaneously injected with 3×10^6^ LNCaP-luc-M6 or PC3M-luc-C6 cells into the dorsal flank. Cells were suspended in 50% matrigel and sterile DBPS in a final volume of 100 µL. All animals were observed daily and tumor volumes were measured at least once a week using a caliper. Tumor volumes were measured according to the formula (π/6) × length^2^ × width. Once tumors were palpable (i.e., a small knot can be felt in the leg), animals were administered 75 mg/kg - 150 mg/kg YK-4-279 daily by IP injection in 20 µL DMSO volumes. In parallel, a control group received DMSO injections. Mice were euthanized if the tumor burden reached 2 cm^3^, if the tumor showed signs of necrosis, or the animals had weight loss greater than 20% of the total body weight. At the end of the study, lung, liver, spleen, kidney, plasma and tumor samples were isolated. Half of each sample was flash frozen and the other half was fixed and stored in 10% formalin. All animal studies were approved by the Georgetown University Institutional Animal Care and Use Committee.

### Analytical Method for Quantitation and Pharmacokinetics of YK-4-279

YK-4-279 was quantitated in plasma or tumor tissue. Tissue homogenates were prepared at a concentration of 200 mg/mL in PBS and further diluted 1∶10 in plasma prior to extraction. YK-4-279 (50 µL of plasma or tissue homogenates) was extracted with 250 µL of acetonitrile containing 0.5 ng/mL of the internal standard NSC668394. After centrifugation, the supernatant was injected into the LC/MS/MS system. Separation was achieved with an Agilent Zorbax XDB-C18 (4.6×50 mm, 5 µm) column at room temperature at 0.4 mL/min for 3 minutes. A gradient was implemented using mobile phases A (water, 0.1% formic acid) and B (acetonitrile, 0.1% formic acid): 40% B for 0.0 to 0.5 min, increased from 40% B to 90% B over 0.5 to 1.5 min, held at 90% B from 1.5 to 2.5 min, and then decreased to 40% B for 2.5 to 3 min for column re-equilibration. To eliminate carryover, an acetonitrile solvent injection was necessary after each sample but was diverted to waste. The analytes were monitored using an AB Sciex triple quadrapole 5500 mass-spectrometric detector (Applied Biosystems, Foster City, CA, USA) using electrospray ionization operating in positive mode. The spectrometer was programmed to allow the [MH+] ions of YK-4-279 and NSC668394 at m/z 368.3 and 452.9, respectively to pass through the first quadrupole (Q1) and into the collision cell (Q2). The daughter ions for YK-4-279 (m/z 135.1) and NSC668394 (m/z 189.2) were monitored through the third quadrupole (Q3). Calibration curves for YK-4-279 was computed using the area ratio peak of the analyte to the internal standard by using a quadratic equation with a 1/x^2^ weighting function over the range of 1 to 2000 ng/mL with dilutions of up to 1∶100 (v∶v).

### Lung Metastasis in Mice

Lung tissues were pulverized into a fine powder by hand grinding using a liquid nitrogen chilled porcelain mortar and pestle. Approximately 30 mg powder was thawed and the remainder stored at -80°C. 200 uL Luciferase cell-culture lysis reagent (Promega) was added to the thawed powder. Samples were vortexed for 15 minutes and subsequently subjected to freeze and thaw cycles 3 times using alternating liquid nitrogen and 37°C water baths. Samples were centrifuged at 10,000 g for 3 min at 4°C. Supernatant was transferred to another tube and the extraction process was repeated without the freeze thaw cycles, after adding another 200 uL lysis buffer to the pellet. The second supernatant was combined with the first. 20 uL of the sample was used to perform a luciferase reading using Luciferase assay system (Promega) according to the manufacturer's protocol.

### In-vitro Luciferase Assay

Cells were plated in 24-well plates. Next day, cells were treated with 1 µM YK-4-279 or DMSO control for 48 hours and lysed using luciferase cell-culture lysis reagent. 20 µL of the sample was used to perform a luciferase reading using Luciferase assay system (Promega) according to the manufacturer's protocol.

### TaqMan Copy Number Reference Assay

Lung metastasis of LNCaP-luc-M6 and PC-3M-luc-C6 xenograft animals were quantified using TaqMan copy number assay protocol (Applied Biosystems). DNA was first extracted from frozen lungs of LNCaP-luc-M6 and PC3M-luc-C6 xenograft animals treated with 150 mg/kg YK-4-279 or vehicle control using Wizard Genomic DNA purification kit according to the manufacturer's protocol (Promega). 100 ng of the extracted DNA was used in each 25 µl qPCR reaction. The number of LNCaP-luc-M6 or PC-3M-luc-C6 metastases were quantified using a human specific primer for the Ribonuclease P RNA component H1 (RPPH1) gene on chromosome 14q11.2. Results were normalized to the amount of mouse tissue present by subtracting Ct values obtained when using mouse specific probe that detects the transferrin receptor gene (TFRC) on chromosome 16qB3.

### Chiral separation of YK-4-279

The chemical and chiral HPLC analyses were performed using Waters XBridge C_18_ (250×4.6 mm) and Chiral Technologies Chiralpak AD (250×4.6 mm) columns, respectively. The enantiomers were resolved by preparative HPLC using a Chiralpak AD column (250 mm×77 mm) packed in-house using a Varian Dynamax Rampak Column Packing Station model 41.4/77. Mobile phase was 60% 2-propanol in heptane and flow rate was maintained at 250 ml/min. All mobile phase batches were premixed by volume. Sample solution was prepared for purification by dissolving the sample in 1∶4∶5 (v/v) dichloromethane/reagent alcohol/heptane. The fractions collected during the purification were transferred to round bottom flasks and evaporated using mild temperature conditions (30–35°C) until all solvent was removed. The analytical HPLC system used for method development and sample analyses was a Waters 2695 Alliance Systems coupled to a Waters 996 Photo Diode (PDA) detector. Preparative HPLC separations were performed using a Waters Delta Prep 2000/4000 equipped with #7 pump heads coupled to a Waters 484 UV-Vis detector.

### Surface Plasmon Resonance Experiments

Direct binding of small molecules to recombinant proteins were measured on a Biacore T200 instrument. CM5 sensor chip surfaces were coated with neutravidin. Biotinylated recombinant ETV1 protein was captured on the surface of the chip by neutravidin-biotin interaction. Protein integrity was confirmed by measuring its binding to wild-type (ATGTAGACCGGAAGTAACTA) and mutant (ATGTAGACCGTAACTA) ETS oligonucleotides in HBS-P Buffer. YK-4-279, R and S enantiomers were screened in buffer containing PBS, 1% DMSO, 0.05% P-20. Data was analyzed using BiaEvaluation software. Data was normalized to molecular weight of compounds.

### Luciferase Assay

Cos-7 cells were co-transfected with a lentiviral plasmid expressing ETV1 mRNA and a vector containing Id2 gene promoter driving expression of a luciferase gene. Transfection was carried out using xtremegene 9 (Roche) according to the manufacturer's protocols. A lentiviral vector expressing LacZ was used as a negative control. Cells were allowed to express ETV1 for 48 hours. Subsequently, they were treated with 10 µM YK-4-279 or enantiomers. Luciferase activity was measured after 24 h using a luciferase assay kit according the manufacturer's protocol (Promega, Madison, WI). Results were normalized to total protein concentration. Statistical analysis was performed using GraphPad Prism 4.0.

### Statistical Analysis

Xenograft tumor growth study used the unpaired t-test with Welch's correction. All statistical tests were two-tailed. *In-vitro* studies were validated in triplicate experiments.

### Ethics Statement

This study was carried out in strict accordance with the recommendations provided by Georgetown University Institutional Animal Care and Use Committee (IACUC). All studies were approved by the IACUC (Protocol Number 11-041). All efforts were made to minimize suffering.

## Supporting Information

Figure S1 **Histopathological analysis of primary tumor samples.** Primary tumors from animals that received 150 mg/kg YK-4-279 were fixed and processed in paraffin blocks. Sections were stained with H&E to evaluate necrotic areas. Neighboring sections were evaluated for cell proliferation by Ki67 immunohistochemistry and for apoptosis by TUNNEL staining.
(TIF)

Figure S2 **Quantification of histological analysis.** Findings from the histopathological analysis are summarized in bar graphs. Primary tumors from five animals in each group are scored for % of tumor area with necrosis, % of cells showing Ki67 staining and % of cells positive for TUNNEL staining. Even though there was a trend for increased necrosis and reduced proliferation in LNCaP cells, data analysis by Student's t test did not show any significant difference between treatment and control groups in any category.
(TIF)

Figure S3 **YK-4-279 does not inhibit luciferase expression.** a) LNCaP-luc-M6 and PC-3M-luc-C6 cells were treated with 1 µM YK-4-279 for 48 hours and the cells were then lysed to perform luciferase assay. YK-4-279 did not affect luciferase expression in LNCaP-luc-M6 and PC3-M-luc-C6 cells. n.s.; not-significant, unpaired student's t-test. b) Primary tumors were harvested from xenograft animals 15 minutes after the last 150 mg/kg compound or vehicle treatment. Protein lysates were obtained from the tissues and used to perform a luciferase assay. Results were normalized to tissue weight. YK-4-279 did not affect luciferase expression in LNCaP-luc-M6 or PC-3M-luc-C6 primary tumors. n.s.; not-significant, unpaired student's t-test.
(TIF)

Figure S4 **PCR quantification of lung metastasis.** Lung metastasis of LNCaP-luc-M6 and PC-3M-luc-C6 xenograft animals treated with 150 mg/kg YK-4-279 or vehicle was quantified using TaqMan copy number reference assay. DNA was extracted from the lungs of these mice and metastasis was quantified with human specific primers for the Ribonuclease P RNA component H1 (RPPH1) gene on chromosome 14q11.2. Results were normalized to the amount of mouse tissue by subtracting Ct values obtained by using a mouse specific probe that detects the transferrin receptor gene (Tfrc) on chromosome 16qB3. Unpaired two-tailed t-test was utilized to assess whether the difference observed was statistically significant. Note: a high ΔCt (Ct_human_-Ct_mouse_) value implies less metastasis burden. **; p<0.005, n.s.; not-significant, unpaired student's t-test.
(TIF)

Table S1 **List of primer sequences used in the study.**
(DOCX)
