# Supplementary figures and images for: A Small Molecule Inhibitor of ETV1, YK-4-279, Prevents Prostate Cancer Growth and Metastasis in a Mouse Xenograft Model
Source: PLoS One. 2014 Dec 5;9(12):e114260. doi: 10.1371/journal.pone.0114260 (PMC4257561; doi:10.1371/journal.pone.0114260)

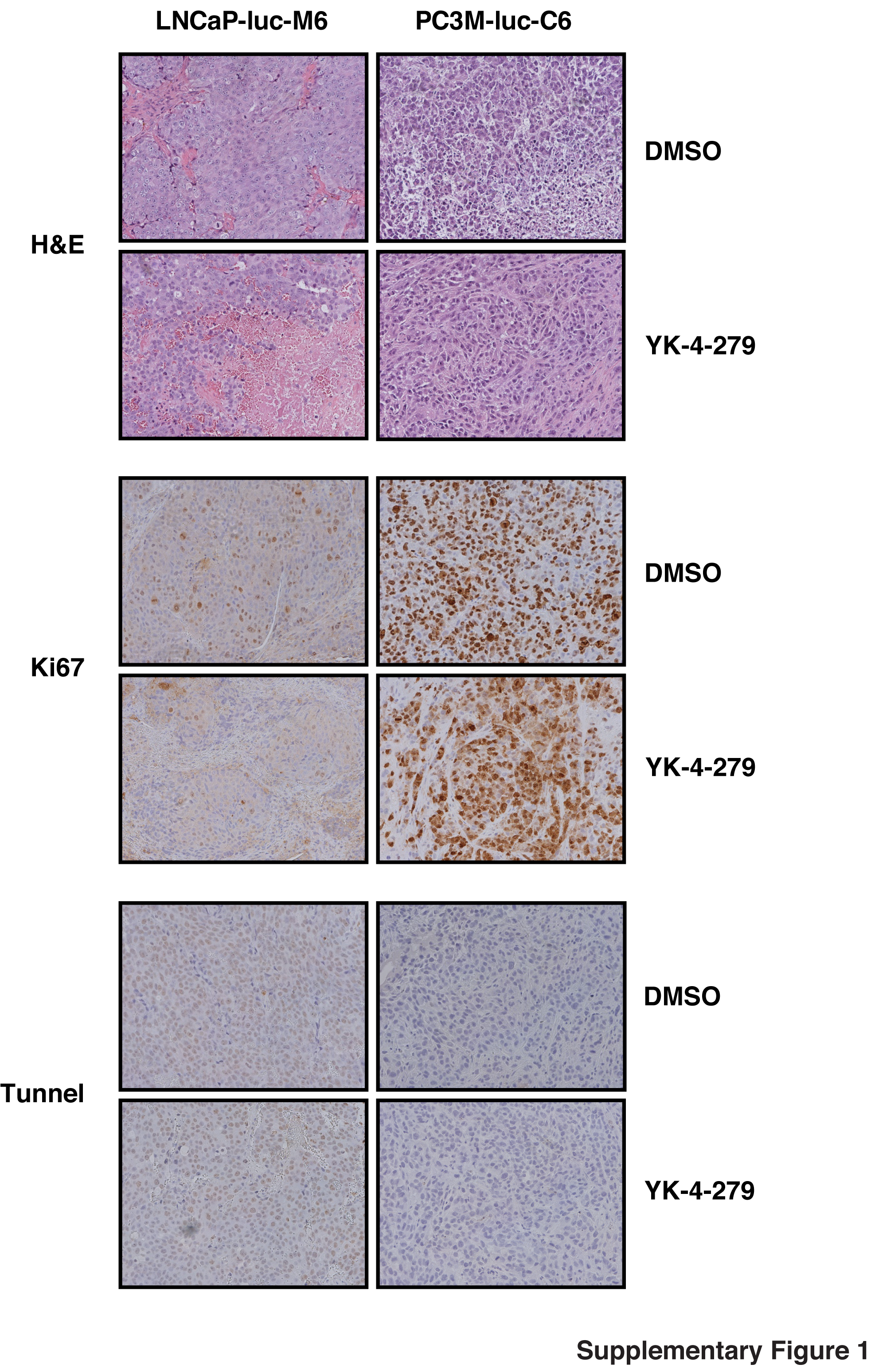

Supplement: Figure S1 — Histopathological analysis of primary tumor samples. Primary tumors from animals that received 150 mg/kg YK-4-279 were fixed and processed in paraffin blocks. Sections were stained with H&E to evaluate necrotic areas. Neighboring sections were evaluated for cell proliferation by Ki67 immunohistochemistry and for apoptosis by TUNNEL staining. (TIF) [file pone.0114260.s001.tif]

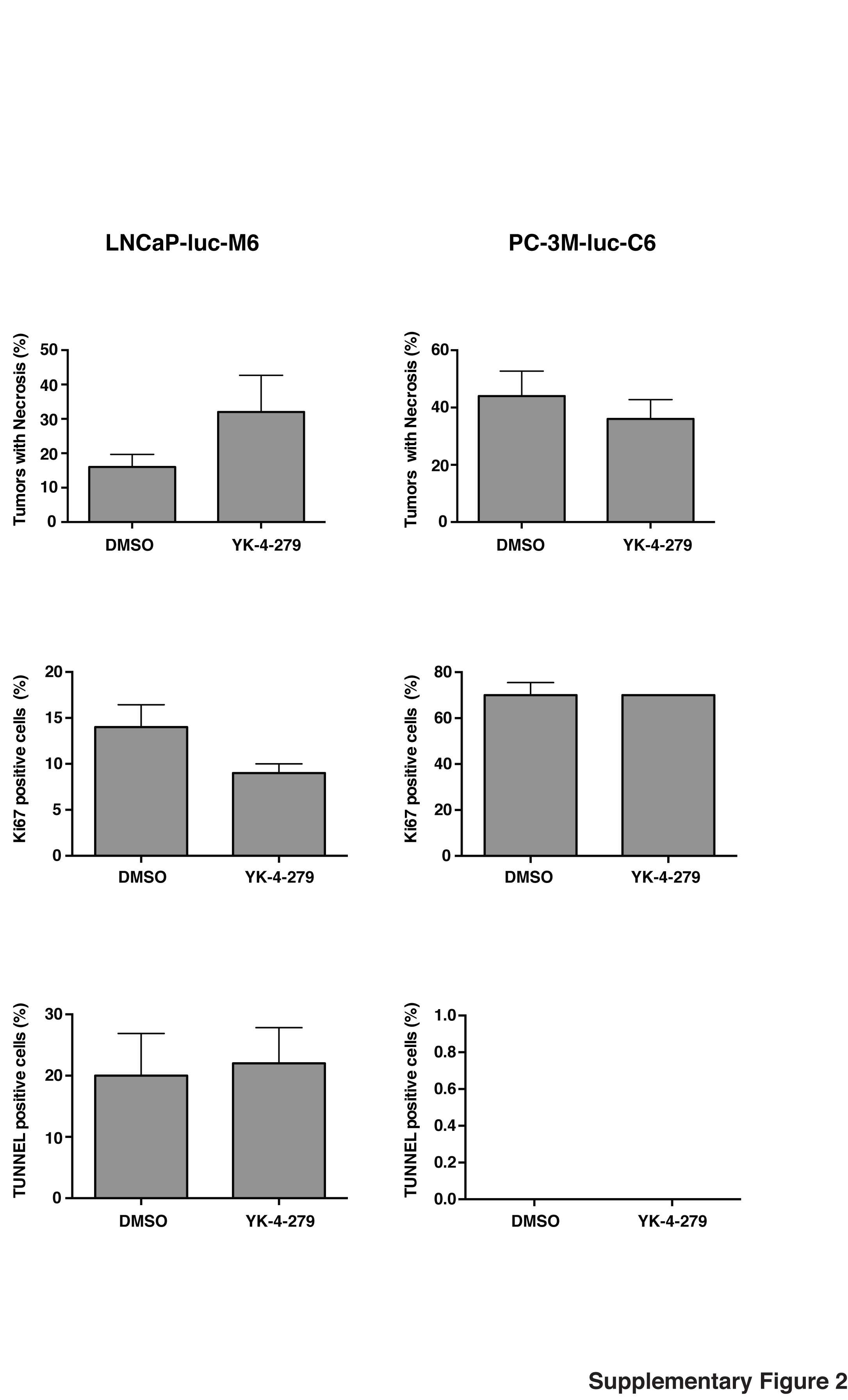

Supplement: Figure S2 — Quantification of histological analysis. Findings from the histopathological analysis are summarized in bar graphs. Primary tumors from five animals in each group are scored for % of tumor area with necrosis, % of cells showing Ki67 staining and % of cells positive for TUNNEL staining. Even though there was a trend for increased necrosis and reduced proliferation in LNCaP cells, data analysis by Student's t test did not show any significant difference between treatment and control groups in any category. (TIF) [file pone.0114260.s002.tif]

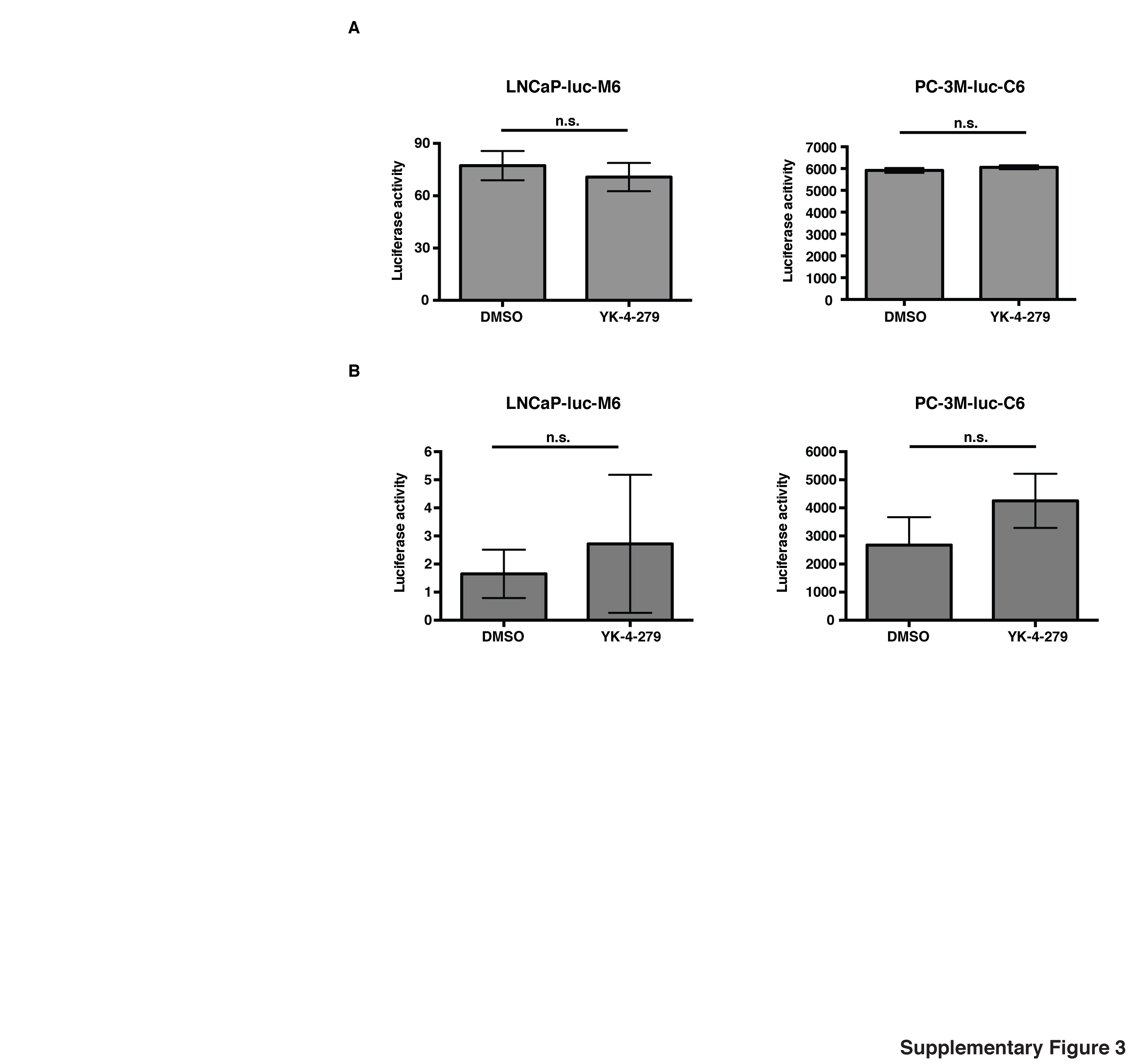

Supplement: Figure S3 — YK-4-279 does not inhibit luciferase expression. a) LNCaP-luc-M6 and PC-3M-luc-C6 cells were treated with 1 µM YK-4-279 for 48 hours and the cells were then lysed to perform luciferase assay. YK-4-279 did not affect luciferase expression in LNCaP-luc-M6 and PC3-M-luc-C6 cells. n.s.; not-significant, unpaired student's t-test. b) Primary tumors were harvested from xenograft animals 15 minutes after the last 150 mg/kg compound or vehicle treatment. Protein lysates were obtained from the tissues and used to perform a luciferase assay. Results were normalized to tissue weight. YK-4-279 did not affect luciferase expression in LNCaP-luc-M6 or PC-3M-luc-C6 primary tumors. n.s.; not-significant, unpaired student's t-test. (TIF) [file pone.0114260.s003.tif]

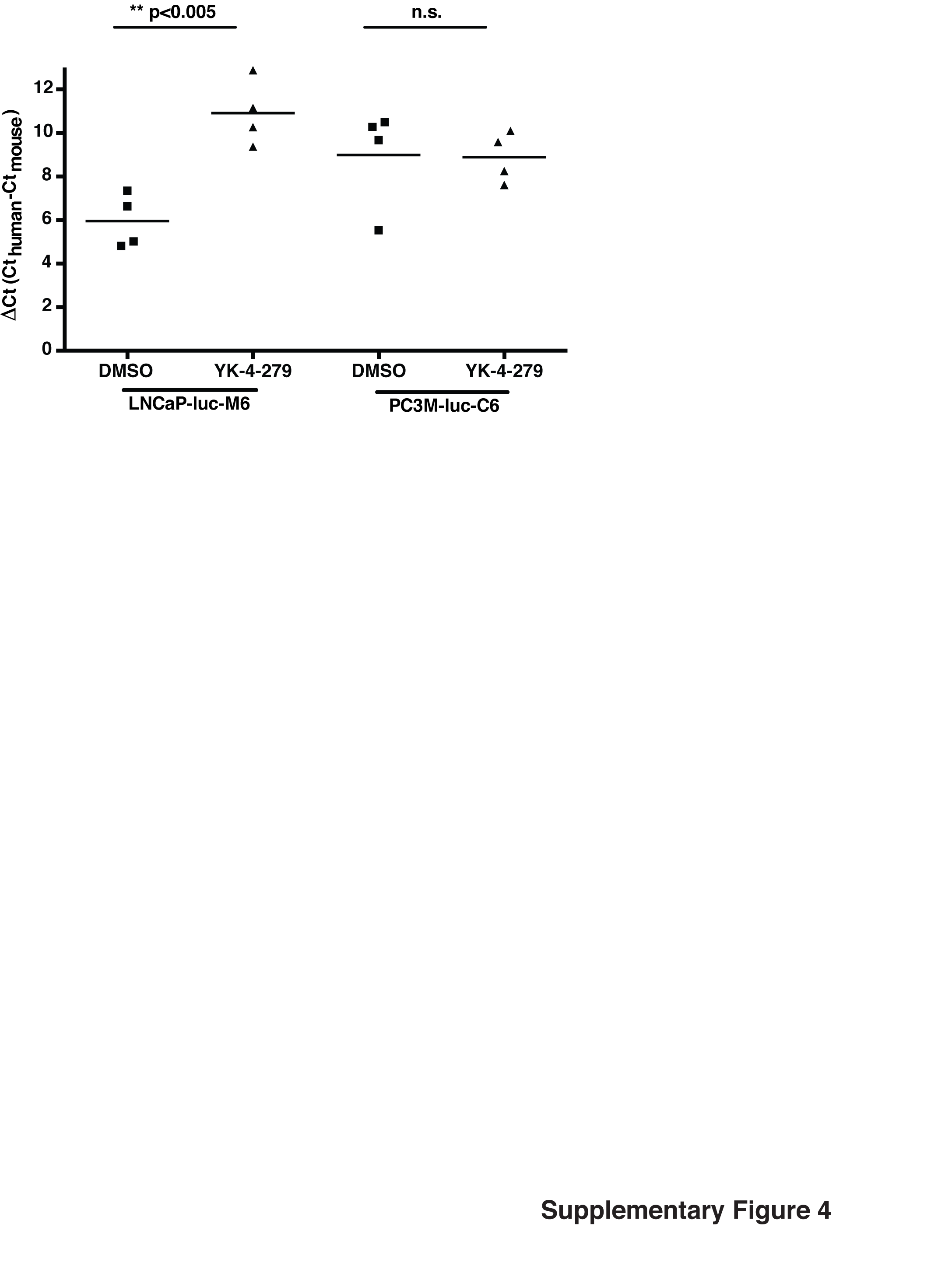

Supplement: Figure S4 — PCR quantification of lung metastasis. Lung metastasis of LNCaP-luc-M6 and PC-3M-luc-C6 xenograft animals treated with 150 mg/kg YK-4-279 or vehicle was quantified using TaqMan copy number reference assay. DNA was extracted from the lungs of these mice and metastasis was quantified with human specific primers for the Ribonuclease P RNA component H1 (RPPH1) gene on chromosome 14q11.2. Results were normalized to the amount of mouse tissue by subtracting Ct values obtained by using a mouse specific probe that detects the transferrin receptor gene (Tfrc) on chromosome 16qB3. Unpaired two-tailed t-test was utilized to assess whether the difference observed was statistically significant. Note: a high ΔCt (Cthuman-Ctmouse) value implies less metastasis burden. **; p<0.005, n.s.; not-significant, unpaired student's t-test. (TIF) [file pone.0114260.s004.tif]
