# Supplementary material for: A Small Molecule Inhibitor of ETV1, YK-4-279, Prevents Prostate Cancer Growth and Metastasis in a Mouse Xenograft Model
Source: PLoS One. 2014 Dec 5;9(12):e114260. doi: 10.1371/journal.pone.0114260 (PMC4257561; doi:10.1371/journal.pone.0114260)
Supplement: Table S1 — List of primer sequences used in the study. (DOCX) [file pone.0114260.s005.docx]

**Primer sequences**

| MMP13-f | TTGAGCTGGACTCATTGTCG |
| --- | --- |
| MMP13-r | GGAGCCTCTCAGTCATGGAG |
| MMP7-f | ATGCGGTGCAAGTCAGCATG |
| MMP7-r | GTCAGGACCGATTGACACGT |
| GLYATL2-f | AGCACTTCAGCTTCTTTCCC |
| GLYATL2-r | ACAGAAACCTTTCCATTGCC |
| FKBP10-f | GGTCCATGCCAGTGATGAG |
| FLBP10-r | AACGGCACTTTTGAAGATGG |
| ETV1-rearrangement-1 | CGCGAGCTAAGCAGGAGGC |
| ETV1-rearrangement-2 | CAGGCCATGAAAAGCCAAACTT |
